# Heterologous Reconstitution of the Intact Geodin Gene Cluster in *Aspergillus nidulans* through a Simple and Versatile PCR Based Approach

**DOI:** 10.1371/journal.pone.0072871

**Published:** 2013-08-23

**Authors:** Morten Thrane Nielsen, Jakob Blæsbjerg Nielsen, Dianna Chinyere Anyaogu, Dorte Koefoed Holm, Kristian Fog Nielsen, Thomas Ostenfeld Larsen, Uffe Hasbro Mortensen

**Affiliations:** Department of Systems Biology, Technical University of Denmark, Kgs. Lyngby, Denmark; University Paris South, France

## Abstract

Fungal natural products are a rich resource for bioactive molecules. To fully exploit this potential it is necessary to link genes to metabolites. Genetic information for numerous putative biosynthetic pathways has become available in recent years through genome sequencing. However, the lack of solid methodology for genetic manipulation of most species severely hampers pathway characterization. Here we present a simple PCR based approach for heterologous reconstitution of intact gene clusters. Specifically, the putative gene cluster responsible for geodin production from *Aspergillus terreus* was transferred in a two step procedure to an expression platform in *A. nidulans*. The individual cluster fragments were generated by PCR and assembled via efficient USER fusion prior to transformation and integration via re-iterative gene targeting. A total of 13 open reading frames contained in 25 kb of DNA were successfully transferred between the two species enabling geodin synthesis in *A. nidulans*. Subsequently, functions of three genes in the cluster were validated by genetic and chemical analyses. Specifically, ATEG_08451 (*gedC*) encodes a polyketide synthase, ATEG_08453 (*gedR*) encodes a transcription factor responsible for activation of the geodin gene cluster and ATEG_08460 (*gedL*) encodes a halogenase that catalyzes conversion of sulochrin to dihydrogeodin. We expect that our approach for transferring intact biosynthetic pathways to a fungus with a well developed genetic toolbox will be instrumental in characterizing the many exciting pathways for secondary metabolite production that are currently being uncovered by the fungal genome sequencing projects.

## Introduction

Fungal natural products constitute a rich resource for bioactive secondary metabolites [1], [2]. To fully exploit this potential, it is essential to identify the genes required for the biosynthesis of these compounds. This process is becoming progressively easier due to the rapidly increasing number of fungal genomes that have been fully sequenced; and since the genes involved in the production of a given secondary metabolite often cluster together in the same region of a chromosome [1]–[3]. Importantly, the genome sequencing projects have revealed that the number of putative gene clusters for secondary metabolite production greatly exceeds the number of known natural products in a given fungus, hence, indicating that most fungal compounds are yet to be discovered.

The prerequisite for genetic exploration of the huge reservoir of undiscovered biosynthetic pathways is solid methodologies for cultivating, propagating and genetically manipulating the producing species. However, the vast majority of newly sequenced organisms fail to meet these requirements, hence, hampering pathway elucidation and exploitation. An attractive solution to this problem is to transfer pathways into another fungus where the methodology is well established. This approach has been used successfully to investigate several gene clusters [4]–[6]. All of these studies apply a strategy where individual genes in a cluster are PCR amplified, cloned, and integrated sequentially into either a random or defined locus. One advantage of this strategy is that it allows easy engineering of individual genes prior to integration in the host strain. In addition, it is possible to insert the foreign gene(s) into a well characterized locus that supports high expression levels [7]. Inserting the genes into a known locus also simplifies strain validation. In a recent example of this strategy, Itoh et al. [6] transferred five genes from *Aspergillus fumigatus* to *A. oryzae* allowing the authors to deduce the biosynthetic route for the meroterpenoid pyropyripene A. However, the strategy may be limited to reconstitution of simple pathways depending on only a small number of genes since assembly of multistep pathways will require several rounds of tedious iterative integration steps or enough genetic markers.

For multistep pathways it is therefore desirable to transfer entire gene clusters from the natural producer to the expression host in one or a few steps. This requires a host, which can efficiently express all genes in the cluster and correctly splice the resulting transcripts. Assembly of multiple genes and PCR fragments can efficiently be performed in *Saccharomyces cerevisiae* by recombination based methods [8]. However, the demand for splicing disfavors *S. cerevisiae* as expression host for this gene transfer strategy as only little splicing occurs in yeast. In contrast, correct and efficient splicing of heterologous transcripts by another filamentous fungus is likely. Activation of the cluster in the new host requires as a minimum that the chromatin structure at the insertion site is in an open configuration and that transcription factors exist that recognize the individual promoters in the cluster. The latter may be facilitated by the fact that many clusters appear to contain one or more genes that encode transcription factors. In a pioneering study, Bergmann et al. showed that expression of such a gene activated the entire aspyridone gene cluster in *A. nidulans* [9]. The potential of transferring entire clusters to an expression host has been demonstrated by Sakai et al. who managed to produce citrinin in *A. oryzae* [10]. To obtain this feat, they isolated and transformed a cosmid from a *Monascus purpureus* library containing all six genes required for production of citrinin into this production host. Here constitutive expression of the citrinin pathway regulator encoded by *ctnA* dramatically increased citrinin production in the heterologous host. However, construction and screening of cosmid libraries is not simple and a versatile PCR based method that facilitates the transfer of entire gene clusters from the natural producer to the expression host is desirable.

We have previously developed a versatile PCR based expression platform that can be used for heterologous expression in *A. nidulans* of one or a pair of genes from the defined locus *IS1*, which supports a high level of gene expression [4], [7]. Here, we demonstrate how this platform can be expanded to allow transfer of an entire gene cluster from another fungus into *IS1*.

## Results and Discussion

### 2.1 Method for PCR based reconstruction of fungal gene clusters in a heterologous host

**Figure 1:**
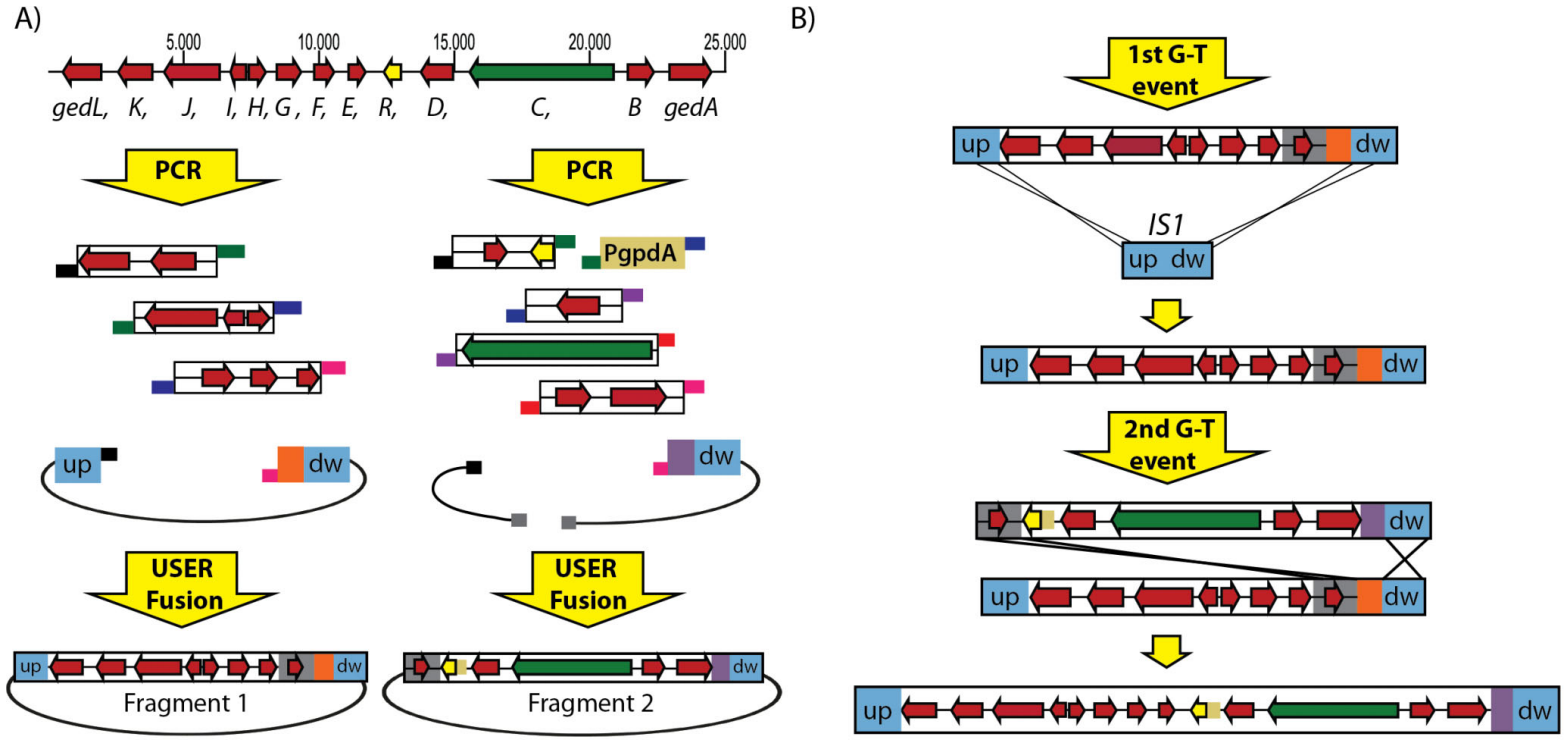
Schematic overview of the PCR based USER cloning strategy for transfer of entire gene clusters from one fungus to another. In the illustrated case, the geodin gene cluster in *A. terreus* is PCR amplified, cloned, and integrated into the *IS1* locus in *A. nidulans*. A) ORFs GedA-GedL are depicted as arrows. The yellow and green arrows represent the ORFs encoding the transcription factor and the PKS, respectively. Remaining ORFs are represented by red arrows. Arrow size is proportional to ORF length and arrow direction indicates genomic orientation. Numbers above the gene cluster specify sequence in base pairs. Genomic DNA fragments and cloning vectors are amplified as PCR products using primers extended with uracil-containing tails. The tails contain matching sequences (indicated by identical colors) allowing for PCR product assembly in a single USER Fusion reaction. For the geodin cluster, all putative ORFs are fused into two fragments, which are individually inserted into a vector prepared for gene targeting. Blue boxes labeled up (upstream) and dw (downstream) represent targeting sequences for homologous recombination into *IS1* in the first gene-targeting event. The targeting sequences in the second integration event are represented in gray and blue and consist of the overlapping region between Fragment 1 and 2 and the downstream part of *IS1*, respectively. Genetic markers used for selection are depicted in orange (*argB*) and purple (*AFpyrG*). The sizes of uracil-containing tails, vector elements and PgpdA fragment are not drawn to scale. B) The first gene-targeting event introduces the first fragment into *IS1* by homologous recombination between *IS1* up and down-sequences as indicated. The second gene-targeting event introduces the second fragment using the overlapping region of the Fragment 1 and 2 (gray) and the downstream section of *IS1* as targeting sequences. Note that additional DNA can be inserted in subsequent gene-targeting events. For example, a third fragment can be inserted by using the downstream end of fragment 2 and the downstream region of *IS1* as targeting sequences. See text for details concerning use and recycling of markers.

Our method for transfer of large DNA fragments relies on successive gene targeting events that introduce ∼15 kb fragments into a defined locus. In the example of the method presented here, we transfer a gene cluster into *IS1* taking advantage of a vector set we have previously developed for this purpose [7]. In our method, fragments covering the entire gene cluster are PCR amplified, combined via USER fusion into ∼15 kb fragments, and inserted into the integration vector by USER cloning (Figure 1A) [11], [12]. The first fragment to be integrated is assembled in the vector and integrated into *IS1* as we have described previously for integration of single genes [7]. The following fragments are integrated as an extension of the previous one by using one of two different markers, *argB* and *pyrG*, for selection. In principle, an indefinite number of integrations can be done, since the marker from the previous integration is excised as the new fragment with the other marker integrates (Figure 1B). This principle is referred to as re-iterative gene targeting [13]. Importantly, marker replacements allows for a simple selection scheme for identification of correctly targeted strains. If, as in our case, the *pyrG* marker is flanked by a direct repeat, we recommend to use the *pyrG* marker in the last integration step, as the *pyrG* marker subsequently can be removed by direct repeat recombination if desirable [14]. In this manner both markers are available in the finalized strain providing a marker repertoire for additional genetic engineering.

**Figure 2:**
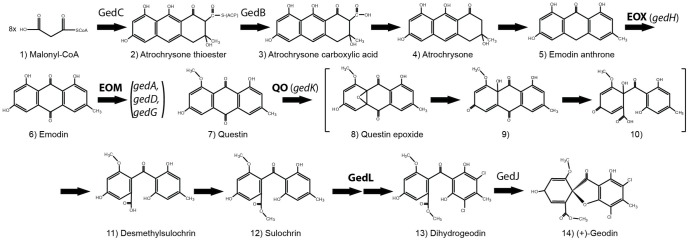
Proposed pathway for geodin production. The PKS (ACTS, [20]), thioesterase (ACTE, [20]) and dihydrogeodin oxidase previously linked to genes as well as the sulochrin halogenase identified in this study (highlighted in bold) are denoted by their *ged*-annotation. Enzymatic reactions for which the enzyme has been characterized but the gene not identified are marked in bold as EOX  =  emodin anthrone oxygenase, EOM  =  emodin-O-methyltransferase and QO  =  questin oxygenase. Reactions involving compounds 8-10 shown in brackets are inferred reactions proposed by Henry and Townsend based on a similar intra-molecular rearrangement in aflatoxin biosynthesis [26], [27].

In the present study we demonstrate the potential of our method by transferring the geodin gene cluster from *A. terreus* to *A. nidulans*. This cluster was chosen, firstly, because it contains a gene encoding a putative transcription factor, which potentially could facilitate activation of the other genes in the cluster. Secondly, the biosynthetic pathway for geodin production is partially characterized (Figure 2) [15]–[21], which simplifies the delineation of the cluster size. Thirdly, the geodin pathway shares several steps with the monodictyphenone pathway including production of several common intermediates/products e.g. emodin [22]. We therefore envisioned, that the chance of producing geodin in *A. nidulans* would be increased, as the corresponding endogenous enzymes could complement geodin enzymes that might not be functional. Moreover, shared intermediates would likely be non-toxic to the host.

### 2.2 Delineation of the putative geodin producing gene cluster in *A. terreus*

Three enzymes involved in geodin production have previously been linked to genes. Specifically, dihydrogeodin oxidase, a polyketide synthase, and a thioesterase are encoded by ATEG_08458 [18], ATEG_08451 [19]–[21], and ATEG_08450 [20], respectively, see *Aspergillus* Comparative Sequencing Project database (Broad Institute of Harvard and MIT, http://www.broadinstitute.org/). As indicated by the gene numbers these genes localize in close proximity to each other strongly suggesting that a gene cluster responsible for production of geodin exists. Three additional enzymes required for geodin biosynthesis have been characterized biochemically *in vitro*: emodin anthrone oxygenase [15], emodin O-methyltransferase [16] and questin oxygenase [17]. Moreover, the occurrence of chlorine atoms in geodin suggests the involvement of a halogenase.

To explore the possibility that genes encoding these four enzymatic activities were also present in this region, we examined all annotated open reading frames (ORFs) positioned between ATEG_08458 and ATEG_08450, as well as 20 kb upstream of ATEG_08458 and downstream of ATEG_08450. Among the ORFs in this region, none had a functional annotation corresponding to these enzymatic activities. We therefore subjected all annotated ORFs in this regions to a functional prediction using the BLAST algorithm [23] from NCBI and the HHpred software [24]. This analysis uncovered three genes that could encode putative methyltransferases (ATEG_08449, ATEG_08452 and ATEG_08456), one ORF that may encode an oxygenase carrying out a Baeyer-Villiger oxidation (ATEG_08459), and a putative halogenase (ATEG _08460), see Table 1.

**Table 1:** Summary of characterized and putative ORFs in the *A. terreus* geodin gene cluster.

BROAD annotation	Proposed annotation	Enzyme class	Validation	Reference
ATEG_08449	GedA	Putative O-methyl transferase*	BLAST, Hhpred	*This study*
ATEG_08450	GedB	β-lactamase type thioesterase	Heterologous expression, in vitro assays	[*20*]
ATEG_08451	GedC	Polyketide synthase	Deletion mutants, heterologous expression, in vitro assays	[*19*]–[*21*]
ATEG_08452	GedD	Putative O-methyl transferase*	BLAST, Hhpred	*This study*
ATEG_08453	GedR	Putative transcription factor	Deletion mutant, quantitative RT-PCR	*This study*
ATEG_08454	GedE	Putative gluthathione-S-transferase	Annotation from BROAD, BLAST, Hhpred	*This study*
ATEG_08455	GedF	Putative oxidoreductase	BLAST, Hhpred	*This study*
ATEG_08456	GedG	Putative SAM-dependent-methyltransferase*	BLAST, Hhpred	*This study*
ATEG_08457-2	GedH	Putative emodin anthrone oxidase, similar to HypC	BLAST, 34% amino acid identity	*This study*
ATEG_08457-1	GedI	Putative mdpH homolog	BLAST, 46% amino acid identity	*This study*
ATEG_08458	GedJ	Dihydrogeodin oxidase	Enzymatic assays, protein sequencing	[*18*]
ATEG_08459	GedK	Putative Bayer Villiger-type oxidase	Enzymatic assays, Identity inferred in this study	[*17*]
ATEG_08460	GedL	Sulochrin halogenase	Deletion mutant, functional complementation	*This study*

All similarity percentages indicate identities at the amino acid level.

* One of these three putative ORFs is likely to encode the emodin O-methyltransferase described by Chen et al [16].

Unexpectedly, none of the annotated ORFs were found to encode the emodin anthrone oxygenase (Figure 2). To investigate this apparent dilemma, we searched the literature for other oxygenases catalyzing a similar reaction. Via this effort, we found an oxygenase that catalyzes conversion of norsolorinic acid anthrone to norsolorinic acid, a step towards aflatoxin production in *A. flavus* [25]. This recently identified enzyme is encoded by the gene *hypC*. Inspired by these findings, we used the sequence of HypC to conduct pair-wise alignments to putative proteins encoded by alternative ORFs in the proposed geodin gene cluster. One short putative ORF encodes a protein of 150 amino acid residues with an overall identity of 34% with the 210 residues of HypC. Moreover, the conserved amino acid residues were primarily positioned in catalytic regions or conserved domains (Figure S1, [25]). Interestingly, the putative ORF is oriented in the opposite direction of ATEG_08457. This strongly indicates that the region at ATEG_08457 is wrongly annotated and contains two separate ORFs that we now denote ATEG_08457-1 (the originally annotated ATEG_08457.1) and ATEG_08457-2 (the new putative *HypC* homolog). Specifically, we suggest that ATEG_08457-2 and ATEG_08457-1 are positioned on *A. terreus* supercontig 12, base pairs 1307175-1307627 and 1308053-1308540, respectively.

Finally, we inspected the remaining ORFs in the region for activities relevant for production of geodin. Among these, one (ATEG_08454) was functionally annotated as a gluthatione-S-transferase and two ORFs (ATEG_08455 and ATEG_08457-1) uncovered by the BLAST analysis displayed similarity to oxidoreductases and MdpH, respectively. The latter is a protein of unknown function required for emodin synthesis in *A. nidulans* [22]. To substantiate our predictions of the involvement of these putative genes, we conferred literature on similar biosynthetic pathways. The requirement for an oxidoreductase in geodin biosynthesis has previously been proposed by Henry and Townsend [26], [27], while Simpson suggested the involvement of both a glutathione-S-transferase and an oxidoreductase in the biosynthesis of xanthones in *A. nidulans* [28].

In addition to genes involved in the biosynthetic steps towards geodin, we noticed the presence of a gene, ATEG_08453, which encodes a putative transcription factor. The position of this gene within the putative geodin gene cluster suggests that it could regulate the activity of all genes in the cluster. In summary, our analysis suggests that the geodin gene cluster spans 25 kb and contains 13 putative ORFs (Figure 1A). Gene numbers, functional predictions and published data resulting from the entire analysis are presented in Table 1.

### 2.3 Strategy for transferring the geodin cluster from *A. terreus* into *A. nidulans*

Two vectors, containing 12 kb (Fragment 1) and 15 kb (Fragment 2) of the putative geodin gene cluster, respectively, were constructed by USER fusion by merging four individual PCR fragments in the first vector and seven PCR fragments in the second (Figure 1A). Assembling the geodin pathway from PCR fragments offers the possibility of introducing defined changes in the DNA sequence prior to integration via the many tools and methods for PCR based genetic engineering. In the present case, we inserted the strong constitutive promoter, *PgpdA*, of *A. nidulans* upstream of ATEG_08453, which encodes the putative transcription factor described above, with the intention that this modification would activate transcription of all genes in the geodin cluster after its integration into *IS1*. Importantly, a fragment of the geodin cluster (2 kb) is included in both constructs to serve as the upstream targeting sequence for the second gene-targeting event as illustrated in Figure 1B.

### 2.4 Production of geodin in *A. nidulans*

**Figure 3:**
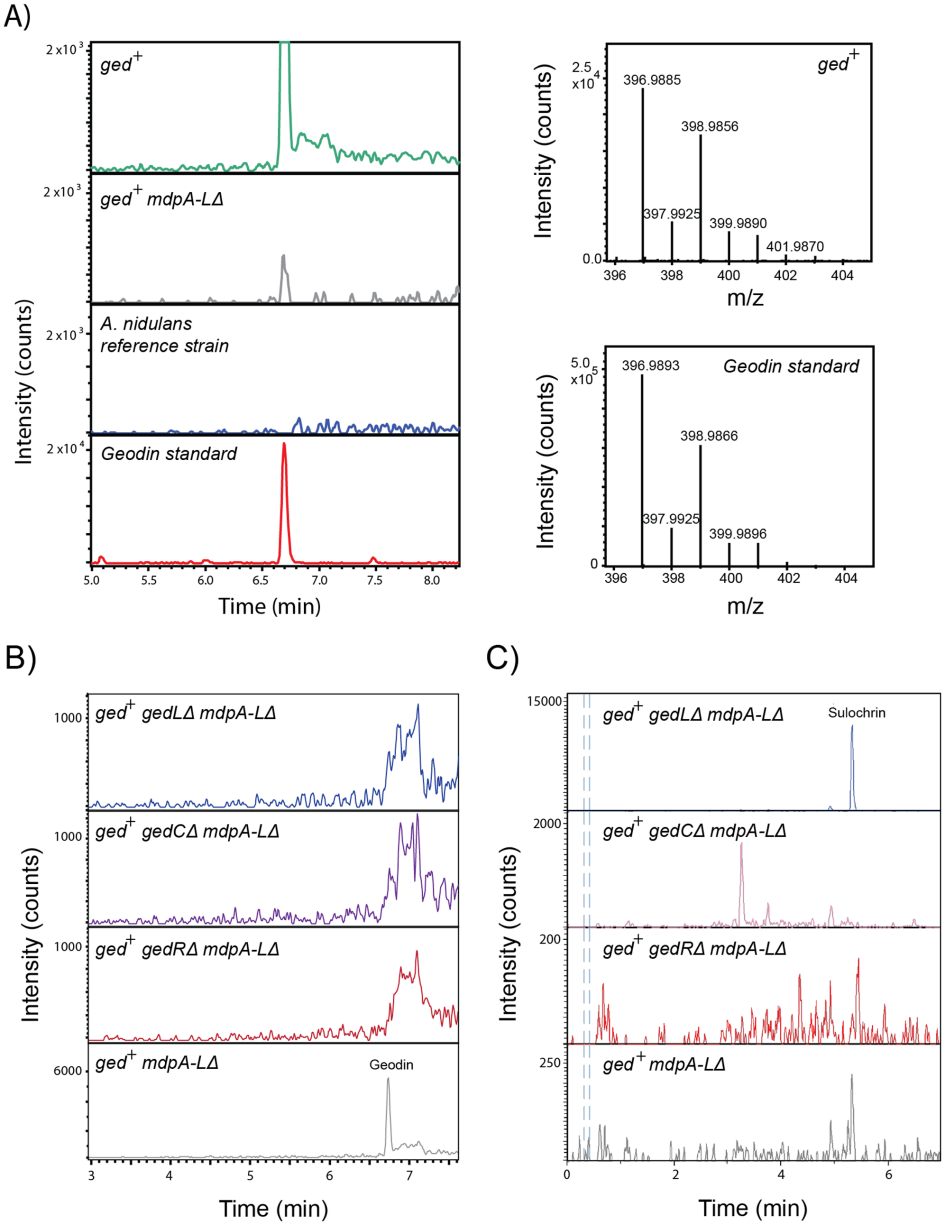
Production of geodin in *A. nidulans ged^+^* strains. **A**) Left panels depict extracted ion chromatograms (ESI-) of geodin *m/z* 396.9876 ± 0.005 amu from fungal extracts of *ged^+^, ged^+^ mdpA-LΔ* and reference strains (Bruker maXis system). An authentic geodin standard is included for comparison. The mass spectra of the putative geodin peak in *ged^+^* and the authentic geodin standard are depicted in panels to the right. **B**) and **C**) ESI^−^ chromatograms of geodin *m/z* 396.9876 ± 0.005 amu (**B**)) and sulochrin *m/z* 331.0812± 0.005 amu (**C**)) extracted from *ged^+^ mdpA-LΔ* (grey), *ged^+^ mdpA-LΔ gedLΔ, (*blue), *ged^+^ mdpA-LΔ gedCΔ* (purple) and *ged^+^ mdpA-LΔ gedRΔ* (red).

Using the two vectors constructed above, the putative geodin gene cluster (*ged*) was successfully transferred to an *A. nidulans* reference strain as well as to a strain where the entire monodictyphenone gene cluster (*mdpA-L*Δ) had been deleted. Geodin production in the *mdpA-L*Δ strains would indicate that all genes in the geodin cluster are functionally expressed, while geodin formation in the reference strain could be mediated via metabolites produced in the monodictyphenone pathway. The ability of the recombinant strains to produce geodin on minimal medium was analyzed by UHPLC-HRMS. The presence of geodin in fungal extracts was identified by comparison of retention time, accurate mass spectra, and isotope ratio to an authentic geodin standard. These analyses demonstrated that both *ged^+^* and *ged^+^ mdpA-L*Δ produced geodin (Figure 3A). Consequently, we suggest that the putative transcription factor, ATEG_08453, is renamed *gedR* and that the enzymes in the cluster (ATEG_08449-08452 + ATEG_08454-08460) are renamed *gedA-L*. In different experiments, we observed that the amount of geodin is reproducibly higher in the *ged^+^* strain (40–70 µg/plate) as compared to the amount in the *ged^+^ mdpA-L*Δ strain (2–4 µg/plate), which does not produce emodin via the *mdp* cluster. We therefore speculate that natively produced emodin, or other intermediates towards emodin production, could be converted into geodin in the *ged^+^*-strains.

### 2.5 Genetic characterization of the geodin cluster in *A. nidulans*

One reason for transferring a gene cluster into a host with a well-developed genetic toolbox is the possibility for further characterization of the cluster. To demonstrate this possibility, we decided to investigate the functionality of three key genes in the cluster, *gedC, gedR* and *gedL* encoding the PKS, the putative regulator, and the putative halogenase, respectively. We focused our efforts on the *ged^+^ mdpA-L*Δ strains, as they provide a genetic background with no risk of complementation by *mdp* enzymes. UHPLC-HRMS analysis of strains grown on minimal medium revealed that all three deletion strains were unable to synthesize geodin (Figure 3B), thereby confirming that geodin is indeed produced from the reconstituted cluster and that the corresponding proteins of all three genes were functional in *A. nidulans* and play a role in geodin biosynthesis. We note the presence of a co-eluting isobaric compound, seen as the broad peak (6.7–7.3 min) in Figure 3B. However, this compound is not geodin as it does not contain a chlorine isotopic pattern. In agreement with previous analyses [19]–[21], no intermediates of the proposed geodin pathway (Figure 2) accumulated in the *ged^+^ gedC*Δ *mdpA-L*Δ strain, which is expected, as the PKS responsible for geodin formation is absent.

According to the proposed biosynthetic route for geodin production, the halogenase accepts sulochrin as substrate and adds two chlorine atoms to form dihydrogeodin (Figure 2). Consistent with the hypothesis that *gedL* encodes the sulochrin halogenase, sulochrin accumulated significantly in the *ged^+^ gedL*Δ *mdpA-L*Δ strain (1.2 – 1.8 µg/plate), but was undetectable in the *gedR* or *gedC* deletion strains (Figure 3C). To confirm that this lack of halogenase activity was due to the *gedL* deletion we reintroduced the *gedL* ORF at another ectopic site, *IS3*, which is a site located on a chromosome different from the one harboring *IS1*, see Figure S2. Surprisingly, no production of geodin was observed in this strain (Figure S3A). This prompted us to perform a BLAST search of the GenBank database [23] using the amino acid sequence of the current ATEG_08460.1 gene model as query. Strikingly, the majority of the best hits were enzymes that contain additional 49 amino acid residues in their N-terminus, including a conserved MSIP/MSVP motif at the very N-terminal end, see Figure S4A. Interestingly, intron prediction based on Augustus [29] predicts an intron just upstream of the AUG proposed by the current gene model (ATEG_08460.1). Taking this into account and by using an ATG further upstream in the *gedL* gene, a very similar extension can be generated for GedL, see Figure S4B and C. We therefore inserted a larger fragment of the *gedL* locus that includes this new ATG as well as its native UTR sequence into *IS2* [4] in the *ged^+^ gedL*Δ *mdpA-L*Δ strain. In this strain, geodin was produced in ample amounts (4.0 – 6.8 µg/plate) strongly suggesting that *gedL* indeed encodes the sulochrin halogenase. Interestingly, in this strain, targeted analysis of the UHPLC-HRMS data and comparison to an in-house metabolite database [30], revealed 0.04 – 0.06 µg/plate of sulochrin and trace amounts of monochlor-sulochrin indicating that chlorine is added in two discrete catalytic steps, see Figure S3B.

**Figure 4:**
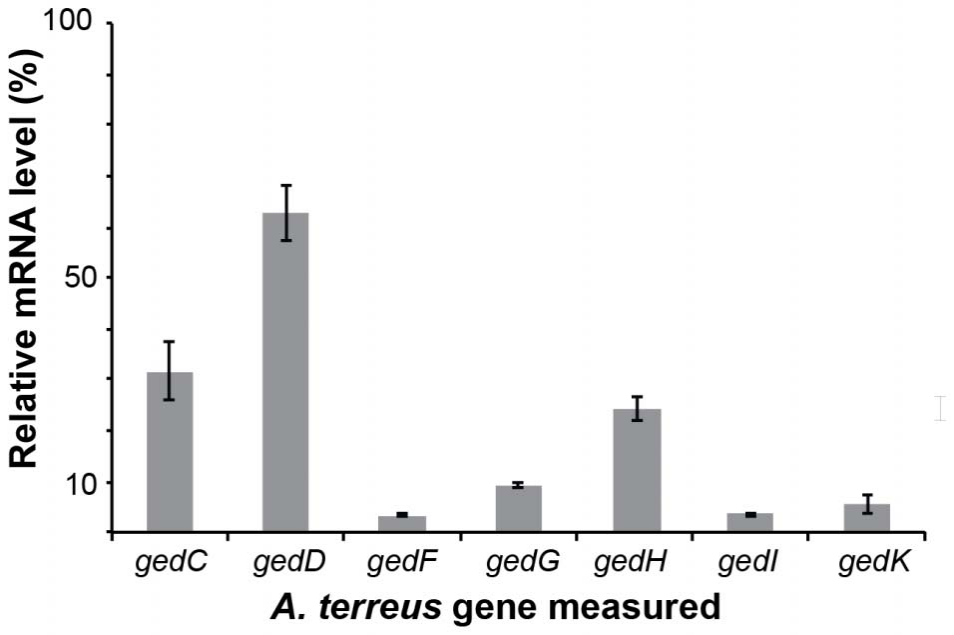
The *A. terreus* transcription factor GedR is important for gene expression in the geodin gene cluster in *A. nidulans*. Transcription levels of selected *ged*-genes in *ged* ^+^ *mdpA-L*Δ, *gedR*Δ strains relative to the corresponding levels in *ged* ^+^ *mdpA-L*Δ strains.

To investigate whether GedR regulates the genes of the geodin cluster in *A. nidulans*, we performed a gene specific mRNA transcript analysis by quantitative RT-PCR in the *ged^+^ mdpA-L*Δ and *ged^+^ gedR*Δ *mdpA-L*Δ strains for all genes in the geodin gene cluster where a putative homolog is present in the monodictyphenone cluster (Table 1). This analysis demonstrated that transcription of all seven selected genes was down regulated in the absence of GedR. Most prominently transcription from four of the genes (*gedF, G, H,* and *K*) was reduced to less than 10% of the level obtained in the *ged^+^ mdpA-L*Δ strain (Figure 4). We note that the de novo annotated candidate gene for the emodin anthrone oxidase, *gedH* (ATEG_08457-2), is transcribed in both the *ged^+^ mdpA-L*Δ and *ged^+^ mdp^+^* strains. In addition, its expression levels in the two strains were different from those obtained for *gedI* (ATEG_08457-1). Together, these observations strongly indicate that *gedR* encodes a transcription factor, which activates the expression of the genes that are involved in geodin synthesis and that *gedH* is a genuine ORF.

Inspired by these results, we next tested whether GedR would activate the *gedR* promoter. To this end we inserted a *lacZ* reporter gene under the control of the native *gedR* promoter into *IS3*, in *ged^+^ mdpA-L*Δ and *ged^+^ gedR*Δ *mdpA-L*Δ strains. On MM medium containing 5-bromo-4-chloro-3-indolyl-β-D-galactopyranoside (X-gal), colonies formed by the *PgpdA-lacZ* positive control strain were strongly blue, see Figure S5. The center of the colonies formed by *ged^+^ mdpA-L*Δ *PgedR-lacZ* strain exhibited slightly blue color. However, this level of blue represents background as it did not differ from the amount and location of blue color produced by the negative control strain *ged^+^ mdpA-L*Δ, see Figure S5. In agreement with this, a quantitative RT-PCR analysis showed that the *lacZ* mRNA level was only modestly increased (1.5 fold) in the *ged* ^+^ *mdpA-L*Δ *PgedR-lacZ* strain as compared to a *ged* ^+^ *ged*RΔ *mdpA-L*Δ *PgedR-lacZ* strain, but this difference was not statistically significant (p = 0.08). Thus, GedR is not sufficient to induce expression from *gedR* in *A. nidulans*.

The fact that geodin production was significantly higher in the *ged^+^* than in the *ged^+^ mdpA-L*Δ strains prompted us to investigate whether GedR could also activate transcription of the *mdp* cluster. Specifically, we compared transcription from *mdpG*, encoding the monodictyphenone PKS, in the *ged^+^* and the reference strains. In agreement with our hypothesis the *mdpG* transcript was easily detectable in the *ged^+^* strain, but undetectable in the reference, see Figure S6.

### 2.6 Conservation of gene clusters resembling the geodin cluster in other fungal species

Finally, we speculated whether gene clusters of a similar organization could be found in other sequenced fungal species as emodin is well-known to serve as precursor to a wide range of natural products [31]–[34]. Comparison of the geodin gene cluster to all Aspergillus genomes available at the *Aspergillus* Comparative Sequencing Project database (Broad Institute of Harvard and MIT, http://www.broadinstitute.org/) revealed the presence of putative gene clusters in *A. fumigatus* and *A. fischerianus* containing putative homologs of 12 of the 13 annotated ORFs in the geodin cluster (the halogenase, *gedL*, is absent). The internal organization of the putative clusters in *A. fumigatus* (Afu4g14450-14580) and *A. fischerianus* (101790-101920) were identical to the geodin cluster with the exception of an inversion affecting the five ORFs *ged*G- *gedK*. Moreover, the amino acid identities between biosynthetic enzymes in *A. terreus* and *A. fumigatus/A. fischerianus* were in average 58% and 60%, respectively. The conservancy across these three species further substantiates our delineation of the geodin cluster and hints that the putative clusters in *A. fumigatus* and *A. fischerianus* may encode the biosynthesis for a similar compound. Both species are known to produce trypacidin [35], which differs from geodin only by the absence of chlorines and the presence of an additional methyl group [36]. In agreement with the structural differences between geodin and trypacidin, the putative *A. fumigatus* and *A. fischerianus* gene clusters contain one additional putative methyltransferase, but lack the putative halogenase. Thus, the two putative clusters are candidates for trypacidin gene clusters.

## Materials and Methods

### Strains and media

*Escherichia coli* strain DH5α was used to propagate all plasmids. Genomic DNA from the geodin producing *A. terreus* IBT15722 strain was used as template for PCR amplification of the geodin cluster. *A. nidulans* strains are shown in Table S1. *A. nidulans* strains were grown on solid glucose minimal medium (MM) prepared as described by Cove [37], but with 1 % glucose, 10 mM NaNO_3_ and 2 % agar. MM was supplemented with 10 mM uridine (Uri), 10 mM uracil (Ura), and/or 4 mM L-arginine (Arg) when required. Solid plates containing 5-fluoroorotic acid (5-FOA) were made as MM+Uri+Ura medium supplemented with filter sterilized 5-FOA (Sigma-Aldrich) to a final concentration of 1.3 mg/ml.

### Vector construction

All vectors were made by USER cloning and USER fusion [7], [11]. All PCR products were amplified in 35 cycles using proof-reading PfuX7 polymerase [38]. Next USER fusions of vector and inserts were performed as previously described [12]. Reactions were incubated for 20 min at 37 °C, followed by 20 min at 25 °C before transformation into *E. coli*.

The pU2111-3 vector was constructed by USER fusion of 5 PCR amplified fragments: 1) vector backbone for propagation in *E. coli* (amplified with primers DH110/DH111), 2) US (upstream) targeting sequence for insertion in *IS3* (DH112/DH113), 3) PgpdA-S::UEC::TtrpC (DH114/DH115), 4) *A. fumigatus pyrG (marker)* (DH116/DH117) and 5) DS (downstream) targeting sequence for insertion in *IS3* (DH118-DH119). UEC: uracil excision cassette. Template for fragments 1 and 3: pU1111, for fragments 2 and 5: *A. nidulans* genomic DNA, and for fragment 4: pDEL2 [13]. p2110-3-*lacZ* was constructed by combing an AsiSI and Nb.BtsI pU2111-3 vector fragment with a PCR product containing the *E. coli lacZ* gene (amplified from pU2110-1-*lacZ* using motni136/motni137 [7] as primers) by USER cloning. The plasmid p2010-3-PgedR-*lacZ* was constructed by USER fusion of 5 PCR amplified fragments: 1) *gedR* promoter sequence (DH120/DH121, template: NID677 genomic DNA with), 2) *lacZ::TtrpC::AFpyrG* (motni136/DH122, template: p2111-3), 3) vector backbone for propagation in E. coli (DH110/DH111 template: pU1111), 4) DS targeting sequence for insertion in *IS3* (DH123/DH119 template: pU2111-3) and 5) US targeting sequence for insertion in *IS3* (DH112/DH113). The vectors pU2110-3-ATEG_08460.1 and pU2110-2-*gedL* were constructed by combining the PCR fragments generated with the primers JBN K35/K36 and JBN W77/W78 with pU2111-3 and pU2111-2 [4] vector fragments, respectively, by USER fusion. Prior to USER fusion both plasmids were digested with AsiSI and Nb.BtsI to create the vector fragments.

The two integration vectors containing the geodin gene cluster, pU1110-1-ged1 (containing Fragment 1) and pU2000-ged2 (containing Fragment 2), were made as follows. Primers for generating all PCR fragments for Fragment 1 and 2 assemblies are shown in Table S2. pU1110-1-ged1 was constructed by combining all relevant fragments for Fragment 1 assembly into an AsiSI and Nb.BtsI pU1111-1 vector fragment by USER fusion. The vector fragment of pU2000-ged2 is based on two PCR fragments generated by using primers 77/422 and 421/70 as well as pJ204 [7] and pU2111-1, respectively, as templates. These two PCR products and all relevant fragments for Fragment 2 assembly were then combined by USER fusion. The inserts in the two integration vectors were fully sequenced (StarSEQ, Germany).

### Construction of A. nidulans strains

Protoplastation and gene-targeting procedures were performed as described by Nielsen et al [14] using either *argB* or AF*pyrG* as marker. All strains were verified by PCR analysis using spores as the source of DNA. Prior to PCR, the samples were incubated for 25 min at 98°C to liberate genomic DNA. This treatment was followed by touch-down PCR programs with annealing temperatures ranging from 64–56°C. Reactions were carried out in 35 cycles using 40 µL volume with less than 1000 spore (one light stab in the colony with a pipette tip).

The *ged^+^* (NID677) and *ged^+^ mdpA-L*Δ (NID695) strains were obtained by transformation of the relevant gene targeting substrates into NID74 [39] or NID356, respectively. The gene targeting substrates containing Fragment 1 and Fragment 2 were liberated from pU1111 (NotI) and pU2052 (SwaI), respectively, and gel purified (GFX^TM^, GE Healthcare) prior to transformation. The resulting strains, NID677 and NID695, were subjected to counter selection on 5-FOA, generating NID802 and NID823, in order to recycle the AF*pyrG* marker, hence, allowing the use of this marker for subsequent gene deletions of *gedC*, *gedL* and *gedR*. These deletions were made as described in [14] using the primers listed in Table S2.

The strains NID1291 and NID1297, expressing *lacZ* under the control of ATEG_08453 promoter was made by transforming a gene targeting substrate liberated from pU2010-3-*PgedR-lacZ* by digestion with SwaI into NID823 and NID925, respectively. A control strain expressing *lacZ* under the constitutive promoter *PgpdA* ¸ NID1278, was constructed by integrating the gene-targeting substrate liberated from pU2110-3-*lacZ* by SwaI. Both gene-targeting substrates integrate into *IS3* located between genes AN4770 and AN4769 on chromosome III, at position 1047840-1051735, by homologous recombination (See Figure S2).

The halogenase complementation strains, ATEG_08460.1 and *gedL*, were made in the NID1279 background, a pop-out recombinant strain from NID843. Digestion of vector pU2110-3-ATEG_08460.1 and pU2110-2-*gedL* liberated gene-targeting constructs for transformation and integration in *IS3* and *IS2*, respectively.

### Chemical characterization of A. nidulans strains

All strains were grown as three point stab inoculations for 7 days at 37°C in the dark on solid MM-media. Extraction and analysis of metabolites were performed by 2 methods: i) The agar plug extraction method described by Smedsgaard [40], using a total of 1 cm^2^ of a colony, followed by analysis using reversed phase separation UHPLC- UV/VIS -HRMS on a maXis G3 quadrupole time of flight (qTOF) mass spectrometer (Bruker Daltonics, Bremen, Germany) connected to an Ultimate 3000 UHPLC system (Dionex, Sunnyvale, CA), and equipped with a 10 cm Kinetex C_18_ column (Phenomenex Torrance, Ca, USA) running a 10–100% acetonitrile gradient system in 10 min at 40°C; ii) more concentrated samples were made by extracting metabolites from a total of 15 cm^2^ of a colony using 12 ml solvent (ethyl acetate-dichloromethane-methanol-formic acid 60∶30∶15∶1 v/v/v) in a 16-ml vial. The extract was evaporated to dryness with N_2_ flow and redissolved in 0.5 ml methanol and analyzed by reversed phase separation an Agilent 1290 UHPLC coupled to an Agilent 6550 qTOF (Santa Clara, CA, USA) equipped with a electrospray source, and equipped with a 25 cm Agilent Poroshell phenyl hexyl running a 10–100% acetonitrile gradient system in 15 min 60°C. Both MS instruments were mainly operated in ESI^−^ as geodin and related compounds ionizes best here in this mode [30]. Identification and quantification of geodin and sulochrin (BioAustralis, Smithfield, NSW, Australia) were based on comparison of peak area, retention time, accurate mass (±1.5 ppm), isotope pattern and adduct pattern to quantitative authentic standards. Non quantitative standards representing 5-O-methylsulochrin; sulochrin-2'-methylether; isosulochrin; 3-O-demethylsulochrin; trypacidin; and emodin were also included in the analyses. Other intermediates were identified by comparison to an internal reference standard database (∼1500 compounds) [30]. For the identification of geodin in NID695, high resolution MS (50 000 FWHM) and mass accuracy (< 1.5 ppm) of the maXis G3 was needed to exclude a non chlorine containing co-eluting isobaric compound, seen as the broad peak in Figure 3 (6.7–7.3 min) that impaired the identification of geodin. In the following strains geodin was further verified by better chromatographic separation on a 25 cm phenyl-hexyl column on the Agilent UHPLC-qTOF.

### RNA isolation and quantitative RT-PCR

RNA isolation from the *A. nidulans* strains and subsequent quantitative RT-PCR reactions were done as previously described in [7] except that biomass for RNA isolation was prepared with a Tissue-Lyser LT (Qiagen) by treating samples for 1 min at 45 MHz. The *A. nidulans* histone 3 encoding gene, *hhtA* (AN0733) was used as an internal standard for normalization of expression levels. All primers used for quantitative RT-PCR are shown in Table S2.

### Bioinformatic analysis of *gedL* (ATEG_08460.1)

Alignment illustrations and sequence were made in CLC Main Workbench 6.8.4.; Alignment parameters Gap open costs  =  10, Gap extension cost  =  1. All sequences in the alignments can be retrieved from Genbank, by the following references for the putative halogenases: *Aspergillus oryzae* ref|XP_001818590.1|, *Aspergillus terreus* ref|XP_001217599.1| and the *A. terreus* genome sequence resource at the Broad Institute, *Bipolaris sorokiniana* gb|EMD66881.1|, *Chaetomium chiversii (RadH)* gb|ACM42402.1|, *Chaetomium globosum* ref|XP_001227515.1|, *Pochonia chlamydosporia* (Rdc2) gb|ADM86580.1| [39] and *Talaromyces stipitatus* ref|XP_002486044.1|. Intron prediction of the *A. terreus* ATEG_08460.1 locus was done based on the Augustus gene prediction resource [29].

## Concluding Remarks

We have described the complete and targeted transfer of all 13 genes of the geodin gene cluster from *A. terreus* to *A. nidulans* through a sequential integration approach enabling *A. nidulans* to synthesize geodin. In principle, this strategy can be used to reconstitute gene clusters of any size as the sequential integrations are based on marker recycling. In addition, defined promoters can easily be introduced in front of relevant genes in the cluster of interest. Importantly, we demonstrate that the cluster can be genetically dissected for clarification of its biochemical potential. We therefore envision that our method will significantly speed up the uncovering of biochemical pathways in fungi where the genome has been sequenced.

## Supporting Information

Figure S1 **Identification of putative HypC homolog encoded by** ***gedH*** **(** ***ATEG_08457-2*** **) in the** ***A. terreus*** **geodin gene cluster.** Pairwise alignment of putative emodin anthrone oxidase, GedH (ATEG_08457-2), from *A. terreus* and norsolinic anthrone oxidase, HypC, from *A. flavus*. The conserved DUF-1772 domain and putative catalytic regions proposed by Ehrlich et al [25] are highlighted in green and blue, respectively.
(TIF)

Figure S2 **Schematic overview of the integration of a gene-expression cassette into the integration site,** ***IS3,*** **by homologous recombination.** *IS3* is located between genes AN4770 and AN4769 on chromosome III. The cassette consists of six parts: upstream targeting sequence (US), promoter (P, in this case 0.5 kb *PgpdA*), your favorite gene (YFG), terminator (T, *TtrpC*), marker (in this case *AFpyrG* flanked by direct), and the downstream targeting sequence (DS). The orientations of the genes AN4770 and AN4769 are indicated by green arrows. The sizes of US, DS and the intergenic region are 1984 bp, 1911 bp, and 3007 bp, respectively.
(TIF)

Figure S3 **Complementation of halogenase deficiency.** **A**) Left panel: detection of sulochrin (-ESI, EIC(*m/z* 331.0812)); right panel: detection of geodin (-ESI, EIC(*m/z* 396.9876)). Strains for halogenase analysis, from top to bottom: NID843 (*gedL*Δ); NID1280 (*gedL*Δ, *IS3::PgpdA-*ATEG_08460.1); and NID1306 (*gedL*Δ, *IS2::gedL*). **B**) Ratio of geodin, monochlor-sulochrin, and sulochrin in the NID1306 strain, including ESI^−^-MS spectrum of monochlor-sulochrin showing the isotopic pattern and the mass deviations relative to the theoretical masses. Reference standards of geodin and sulochrin were included in all runs (data not shown).
(TIF)

Figure S4 **Identification of the likely start codon of** ***gedL*** **.** **A**) Alignment of the top hits in a BLAST search for ATEG_08460.1 homologs shows that they contain a very conserved 48 amino acid residue addition in the N-terminus. Amongst the homologs, Rdc2, has been characterized as a halogenase by [39] MLAS is the predicted N-terminus of ATEG_08460.1. Drawing is not to scale. **B**) The position of putative exons and intron in the 5’end of *gedL* as predicted by the Augustus software [29]. The predicted protein sequence encoded by exon 1 and by the first section of exon 2 is indicated. **C**) Full alignment of the halogenase homologs and GedL based on the GedL sequence derived from the new start codon.
(TIF)

Figure S5 **Expression of** ***lacZ*** **under the control of the** ***gedR*** **promoter (** ***PgedR*** **).** Left panel: the positions of the strains on the plate are shown in the right panel. NID823 (*ged^+^ mdpA-L*Δ) is the reference strain without the *lacZ* gene. NID1278 is a control strain containing the *PgpdA-lacZ* construct in *IS3*. The NID1291 (*ged^+^ mdpA-L*Δ *PgedR-*lacZ) strain carries *PgedR-*lacZ in *IS3.* The strains were stabbed on MM containing X-gal and incubated three days at 37 °C in the dark before photography.
(TIF)

Figure S6 **Constitutive expression of** ***gedR*** **induces transcription of the** ***A. nidulans*** **gene** ***mdpG*** **.** *mdpG* mRNA levels in reference (NID1) and in the *ged^+^* strain (NID677) were evaluated by quantitative RT-PCR. For each strain, RNA was extracted as described in Materials and Method and the RNA samples analyzed in triplicate by quantitative RT-PCR. The samples were loaded and analyzed by 1% agarose gel-electrophoresis as indicated in the figure.
(TIF)

Table S1 **Strain genotypes.** * =  For reference see Nielsen et al ([13]).
(XLSX)

Table S2 **Primers list.** The sequence of PCR products # 1-3 corresponds to supercontig 12: 1302695-1315163 (ATEG_08454-08460) in the genome sequenced isolate NIH2624 (IBT28053). Similarly, PCR products # 4-6 and 8 corresponds to supercontig 12: 1289727-1304484 (ATEG_08449-08454) in NIH2624 (IBT28053). * =  For reference see Hansen et al ([7]).
(XLSX)
