# Correction: Heterologous Reconstitution of the Intact Geodin Gene Cluster in *Aspergillus nidulans* through a Simple and Versatile PCR Based Approach

**DOI:** 10.1371/annotation/c05c3fd1-dd00-4840-891d-693c614aaaf9

**Published:** 2013-11-08

**Authors:** Morten Thrane Nielsen, Jakob Blæsbjerg Nielsen, Diana Chinyere Anyaogu, Dorte Koefoed Holm, Kristian Fog Nielsen, Thomas Ostenfeld Larsen, Uffe Hasbro Mortensen

The third author was incorrectly indicated as a Corresponding Author of the article. This author should instead be noted as having contributed to the article equally with the first two authors.

In addition, the third author's name was spelled incorrectly. The correct name is: Diana Chinyere Anyaogu. 

