# Supplementary figures and images for: Heterologous Reconstitution of the Intact Geodin Gene Cluster in Aspergillus nidulans through a Simple and Versatile PCR Based Approach
Source: PLoS One. 2013 Aug 23;8(8):e72871. doi: 10.1371/journal.pone.0072871 (PMC3751827; doi:10.1371/journal.pone.0072871)

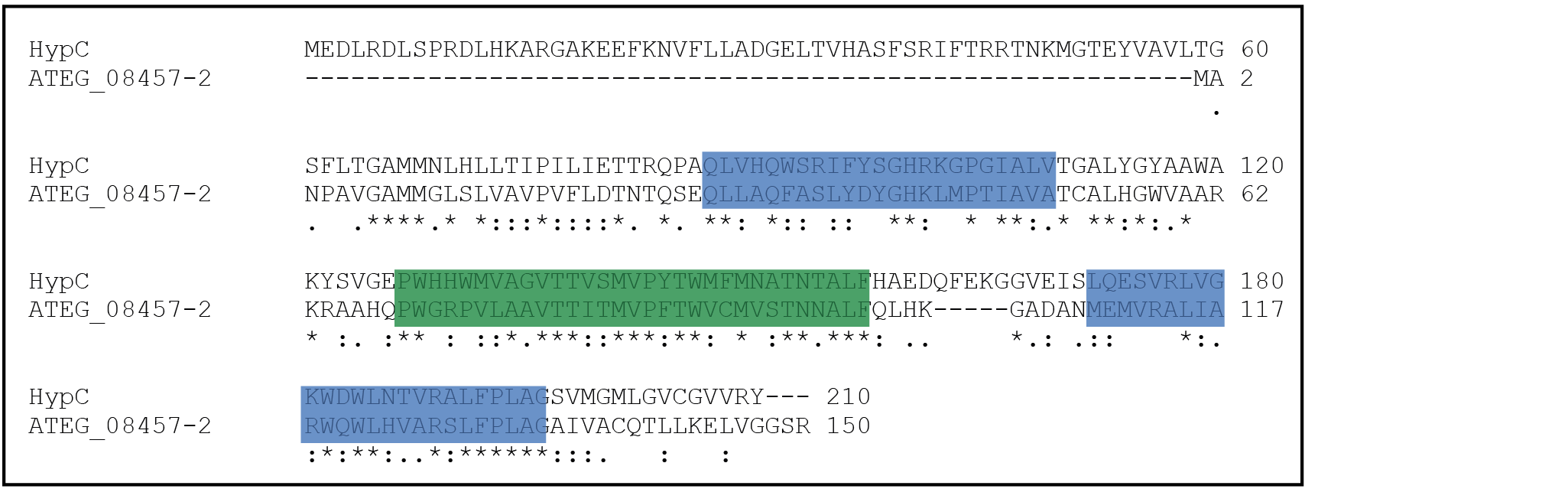

Supplement: Figure S1 — Identification of putative HypC homolog encoded by gedH ( ATEG_08457-2 ) in the A. terreus geodin gene cluster. Pairwise alignment of putative emodin anthrone oxidase, GedH (ATEG_08457-2), from A. terreus and norsolinic anthrone oxidase, HypC, from A. flavus. The conserved DUF-1772 domain and putative catalytic regions proposed by Ehrlich et al [25] are highlighted in green and blue, respectively. (TIF) [file pone.0072871.s001.tif]

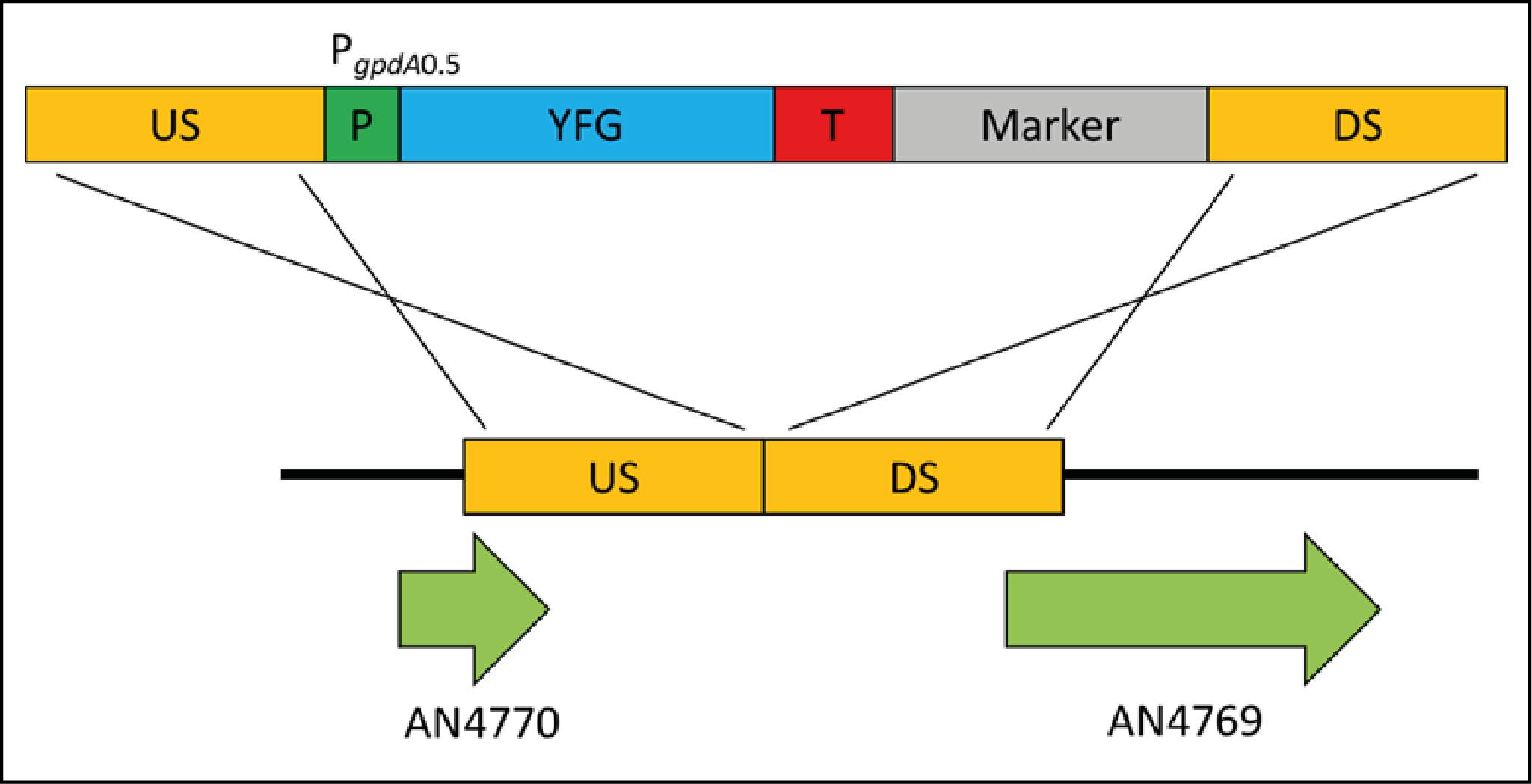

Supplement: Figure S2 — Schematic overview of the integration of a gene-expression cassette into the integration site, IS3, by homologous recombination. IS3 is located between genes AN4770 and AN4769 on chromosome III. The cassette consists of six parts: upstream targeting sequence (US), promoter (P, in this case 0.5 kb PgpdA), your favorite gene (YFG), terminator (T, TtrpC), marker (in this case AFpyrG flanked by direct), and the downstream targeting sequence (DS). The orientations of the genes AN4770 and AN4769 are indicated by green arrows. The sizes of US, DS and the intergenic region are 1984 bp, 1911 bp, and 3007 bp, respectively. (TIF) [file pone.0072871.s002.tif]

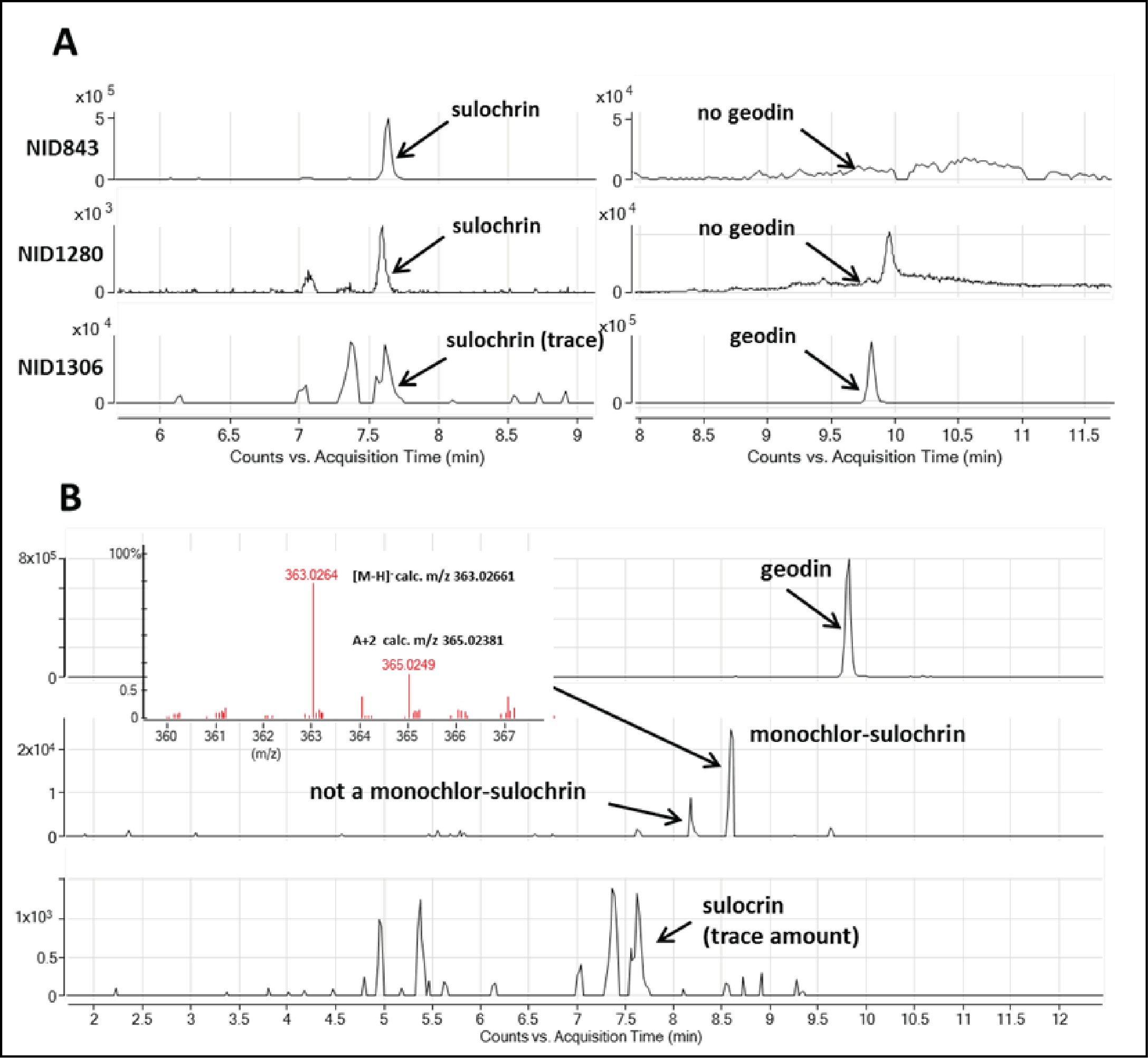

Supplement: Figure S3 — Complementation of halogenase deficiency. A) Left panel: detection of sulochrin (-ESI, EIC(m/z 331.0812)); right panel: detection of geodin (-ESI, EIC(m/z 396.9876)). Strains for halogenase analysis, from top to bottom: NID843 (gedLΔ); NID1280 (gedLΔ, IS3::PgpdA-ATEG_08460.1); and NID1306 (gedLΔ, IS2::gedL). B) Ratio of geodin, monochlor-sulochrin, and sulochrin in the NID1306 strain, including ESI−-MS spectrum of monochlor-sulochrin showing the isotopic pattern and the mass deviations relative to the theoretical masses. Reference standards of geodin and sulochrin were included in all runs (data not shown). (TIF) [file pone.0072871.s003.tif]

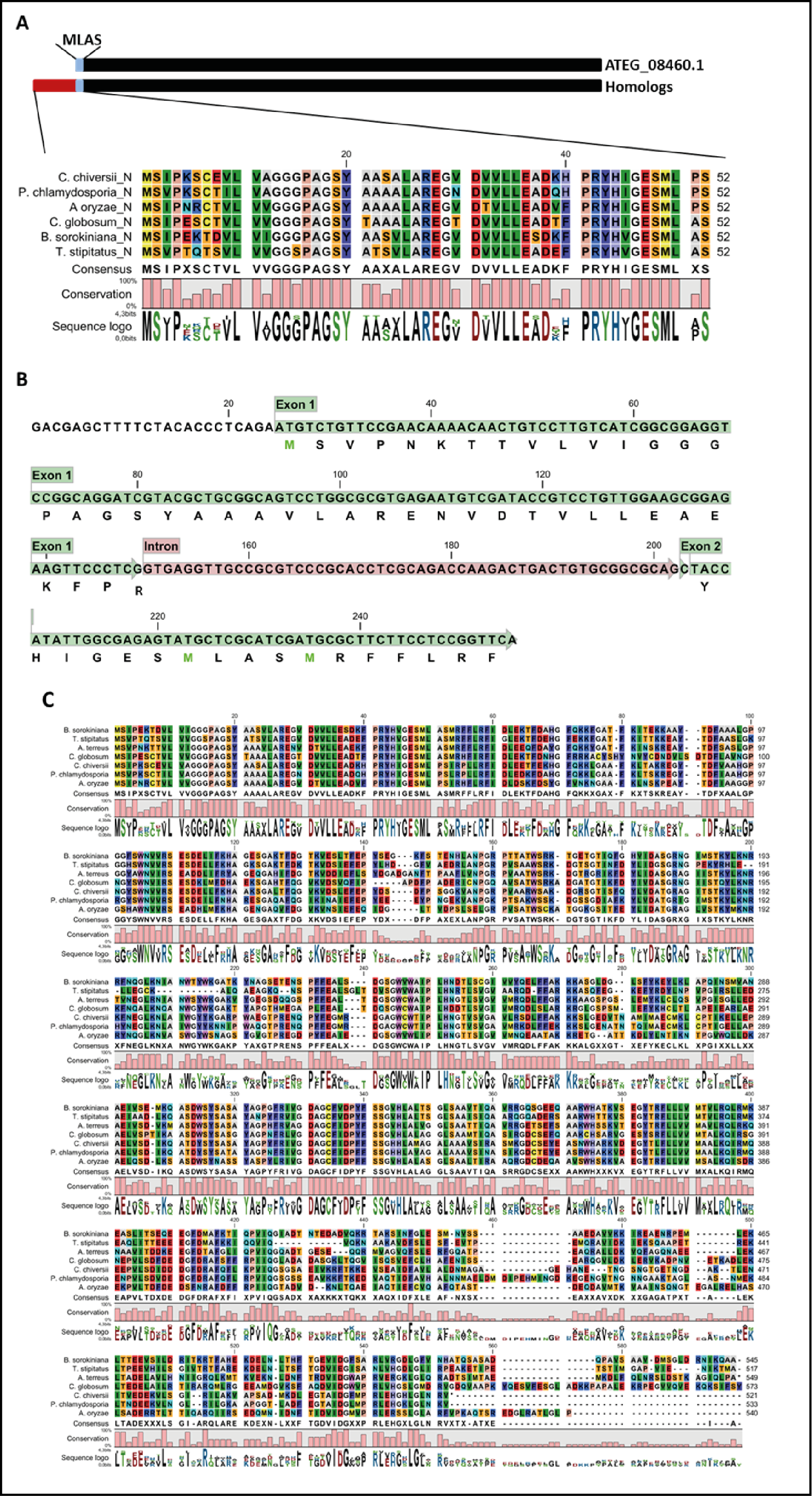

Supplement: Figure S4 — Identification of the likely start codon of gedL . A) Alignment of the top hits in a BLAST search for ATEG_08460.1 homologs shows that they contain a very conserved 48 amino acid residue addition in the N-terminus. Amongst the homologs, Rdc2, has been characterized as a halogenase by [39] MLAS is the predicted N-terminus of ATEG_08460.1. Drawing is not to scale. B) The position of putative exons and intron in the 5’end of gedL as predicted by the Augustus software [29]. The predicted protein sequence encoded by exon 1 and by the first section of exon 2 is indicated. C) Full alignment of the halogenase homologs and GedL based on the GedL sequence derived from the new start codon. (TIF) [file pone.0072871.s004.tif]

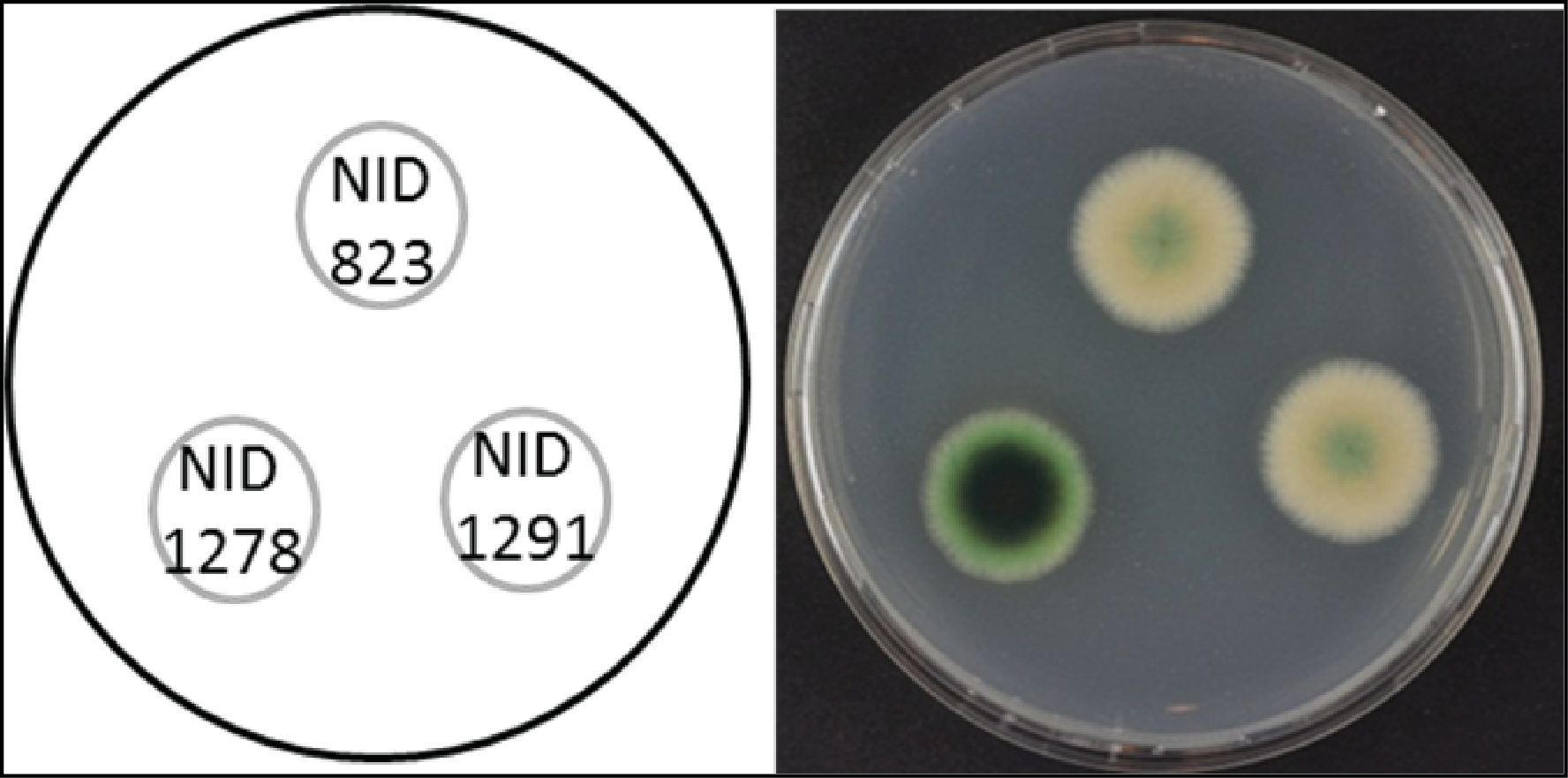

Supplement: Figure S5 — Expression of lacZ under the control of the gedR promoter ( PgedR ). Left panel: the positions of the strains on the plate are shown in the right panel. NID823 (ged+ mdpA-LΔ) is the reference strain without the lacZ gene. NID1278 is a control strain containing the PgpdA-lacZ construct in IS3. The NID1291 (ged+ mdpA-LΔ PgedR-lacZ) strain carries PgedR-lacZ in IS3. The strains were stabbed on MM containing X-gal and incubated three days at 37 °C in the dark before photography. (TIF) [file pone.0072871.s005.tif]

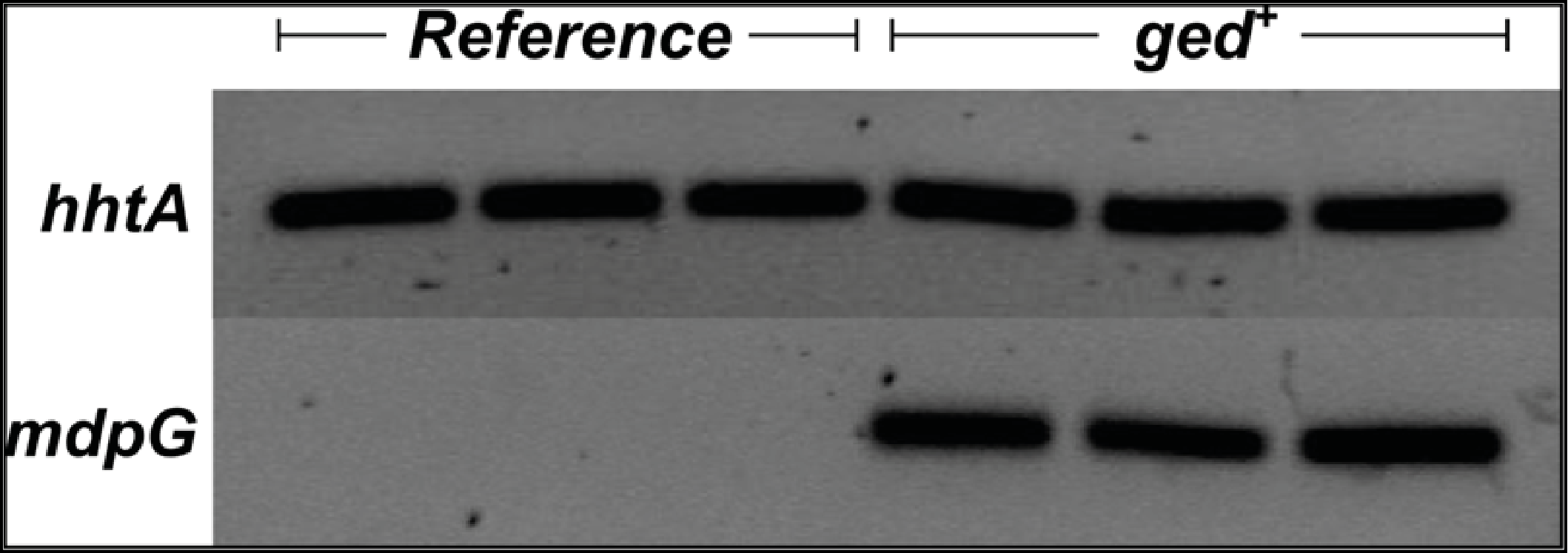

Supplement: Figure S6 — Constitutive expression of gedR induces transcription of the A. nidulans gene mdpG . mdpG mRNA levels in reference (NID1) and in the ged+ strain (NID677) were evaluated by quantitative RT-PCR. For each strain, RNA was extracted as described in Materials and Method and the RNA samples analyzed in triplicate by quantitative RT-PCR. The samples were loaded and analyzed by 1% agarose gel-electrophoresis as indicated in the figure. (TIF) [file pone.0072871.s006.tif]
